# Distinguishing Stoichiometric Homeostasis of Soil Microbial Biomass in Alpine Grassland Ecosystems: Evidence From 5,000 km Belt Transect Across Qinghai–Tibet Plateau

**DOI:** 10.3389/fpls.2021.781695

**Published:** 2021-12-02

**Authors:** Jihui Fan, Tianyuan Liu, Ying Liao, Yiying Li, Yan Yan, Xuyang Lu

**Affiliations:** ^1^Institute of Mountain Hazards and Environment, Chinese Academy of Sciences, Chengdu, China; ^2^Key Laboratory of Ecosystem Network Observation and Modelling, Synthesis Research Centre of Chinese Ecosystem Research Network, Institute of Geographic Sciences and Natural Resources Research, Chinese Academy of Sciences, Beijing, China; ^3^College of Resources and Environment, University of Chinese Academy of Sciences, Beijing, China

**Keywords:** Qinghai–Tibet Plateau (QTP), alpine grassland, soil microbe, stoichiometric homeostasis, trade-off

## Abstract

The biogeographic characteristics of soil microbial biomass stoichiometry homeostasis and also its mechanisms are commonly thought to be key factors for the survival strategies and resource utilization of soil microbes under extreme habitat. In this work, we conducted a 5,000-km transect filed survey in alpine grassland across Qinghai–Tibet Plateau in 2015 to measure soil microbial biomass carbon (MBC) and nitrogen (MBN) across alpine steppe and meadow. Based on the differences of climate and soil conditions between alpine steppe and meadow, the variation coefficient was calculated to investigate the homeostatic degree of MBC to MBN. Furthermore, the “trade-off” model was utilized to deeply distinguish the homeostasis degree of MBC/MBN between alpine steppe and meadow, and the regression analysis was used to explore the variability of trade-off in response to environmental factors in the alpine grassland. The results showed that the coefficient of variation (CV) of MBC/MBN in alpine meadow (CV = 0.4) was lower than alpine steppe (CV = 0.7). According to the trade-off model, microbial turnover activity of soil N relative to soil C increased rapidly and then decreased slightly with soil organic carbon (SOC), soil total nitrogen (STN), and soil water content across alpine meadow. Nevertheless, in alpine steppe, SOC/STN had a positive effect on microbial turnover of soil N. These results suggested that water, heat, and soil nutrients availability were the key factors affecting the C:N stoichiometry homeostasis of soil microbial biomass in Qinghai–Tibet Plateau (QTP)’s alpine grassland. Since the difference of survival strategy of the trade-off demands between soil C and N resulting in different patterns and mechanism, the stoichiometry homeostasis of soil microbial biomass was more stable in alpine meadow than in alpine steppe.

## Introduction

Ecological stoichiometry focuses on the balance and limitation of energy and multiple chemical elements in various ecosystems (Agren and Weih, 2020; Zhang et al., 2020), involving competition, herbivory, mutualism, food webs, and biogeochemistry (Sterner and Elser, 2002; Zhou et al., 2021). This theory can be quantified to research on the relationship between biology and environment, biological interaction, and biogeochemical cycle during the process of energy flow and material cycle (Crous et al., 2019; Wang et al., 2021; Zhou et al., 2021). The scales ranged from genes to biosphere (Wang et al., 2015; Cherif et al., 2017; Tian et al., 2018) and can be unified (Zeng and Chen, 2005) to reveal ecological structure, function, process, and stability (Fang et al., 2019; Li et al., 2021; Wang et al., 2021). Numerous studies have demonstrated the stoichiometric homeostasis among various living organisms (Elser et al., 2000; Sterner and Elser, 2002), but with large variations among ecological studies (McGroddy et al., 2004; Elser et al., 2010; Giordano, 2013).

The homeostasis of biological stoichiometry emphasizes that living organisms can maintain relatively stable and balanced state of its multiple elements’ composition in the changing environment (Sterner and Elser, 2002). The theory of stoichiometry and its homeostasis reflect the requirements for constructure and metabolism of organisms (Michaels, 2003; Allen and Gillooly, 2009). The Redfield ratio, which describes the proportions of elements equal to 106 atoms of C per 16 atoms of N per 1 atom of P for marine organisms in ocean water (Redfield, 1958). Nevertheless, the biological characteristics of stoichiometric homeostasis might change with different ontogenetic stages (Wang et al., 2021), stoichiometric variation of their diet (Hood and Sterner, 2010; Wang et al., 2018), and various types of species (e.g., weak homeostasis of phototrophs is opposite to strong homeostasis of heterotrophs) (Sterner and Elser, 2002). Furthermore, stoichiometric homeostasis reflects biological adaptation by biochemical and physiological adjustments of multiple elements that respond to living environment (Elser et al., 2010; Li et al., 2021). It can be an indicator of ecosystem dynamics (Guo et al., 2017) and the organism’s ability to maintain consistent or stable state (Meunier et al., 2014) facing environmental fluctuations. Consequently, there have existed various geographical scales of its research including individual (Abail and Whalen, 2018), population (Andersen et al., 2004), community (Fanin et al., 2017), ecosystem (Yu et al., 2010; Wang et al., 2021), national (Zhang et al., 2018), and global (Galbraith and Martiny, 2015) scale, and it has been also explored in forest (Bai et al., 2019), wetland (Julian et al., 2020), grassland (Yu et al., 2011; Dijkstra et al., 2012), and aquatic (Gong et al., 2018) ecosystems.

However, the relationship of stoichiometric homeostasis with biogeochemical processes in the soil which is the main place for the life activity (Chapin et al., 2011) and ecosystem exchange of material and energy (Li et al., 2004) remains limited (Wang et al., 2014; Feng and Bao, 2017). Particularly, soil microorganism is the main component of soil biology and the core indicator of material cycling and energy flow in the soil ecosystem (Pascual et al., 2000; Rogers and Tate, 2001). Moreover, soil microorganisms are very sensitive to changes of environmental factors so that it can reflect the changes of soil quality status and ecosystem function earlier (Caravaca et al., 2003; Chapin et al., 2011). Previous studies have focused on the C:N:P atomic ratio of soil microbial biomass across global scale and found that the ratio is relatively consistent, with the value of 60:7:1 (Cleveland and Liptzin, 2007) or 42:6:1 (Xu et al., 2013), and C:N ratios of microbes vary from 8:1 to 12:1, which is also relatively consistent (Wright and Coleman, 2000). Further, elementary ratios might vary significantly in different habitats (McGroddy et al., 2004). Meanwhile, soil microbes can adjust their stoichiometry of elements when the supply of multiple resources is imbalanced (Fanin et al., 2017). These studies improved our understanding about stoichiometric homeostasis of soil microbes, but the stoichiometry homeostasis characteristics of soil microbial communities in the extreme habitat (such as alpine ecosystem), which is extremely sensitive to environmental changes, or not (Chapin et al., 2008), is largely unknown.

Qinghai–Tibet Plateau (QTP) is the amplifier to respond global change (Liu et al., 2019) due to its unique alpine climate and harsh environment (Sun et al., 2019). QTP’s ecosystem is dominated by alpine grasslands (alpine meadow and steppe) which cover more than 60% of QTP area (Wu et al., 2011). Notably, the spatial distribution of water, nutrient, and heat availability over the QTP is heterogeneous. For instance, there is a decreasing gradient of the precipitation and soil nutrient content from the southeast to the northwest of QTP (Yang et al., 2008; Tan et al., 2010; Zhou et al., 2020), and alpine meadow and steppe are separately distributed at the relatively humid and arid circumstance (Qin et al., 2018; Zhou et al., 2020). All these characteristics imply that soil microorganisms may take various strategies to adjust their homeostasis to adapt to sensitive and heterogeneous habitats of alpine grasslands on QTP.

Here, we conducted a belt-transect survey across alpine meadow and steppe (5,000 km) across QTP, with measurements of soil microbial biomass carbon (MBC) and nitrogen (MBN). These data, together with climatic and edaphic other factors, were used to test the overarching hypothesis that the stoichiometric homeostasis of soil microorganisms is moderated by soil nutrient, availability, and also hydrothermal condition. Furthermore, we untangle the underlying mechanisms on how water, heat, and nutrient availability mediate stoichiometric homeostasis of soil microbes to adapt alpine habitat.

## Materials and Methods

### Study Area

**FIGURE 1:**
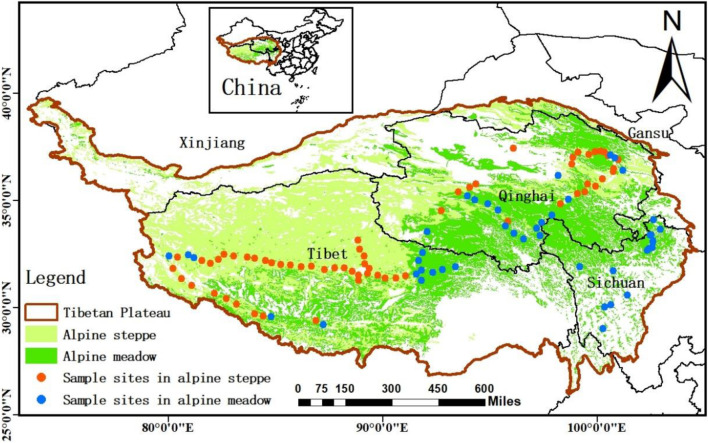
The study area and sample sites. The sample sites (108 sites) were natural zonal grasslands across Qinghai–Tibet Plateau, including alpine meadow and alpine steppe grasslands.

Our sampling sites covered Qinghai–Tibet Plateau, which is viewed as the “Third Pole” and “Asia’s water tower” (average elevation is about 4,000 m) (Sun et al., 2019; Xiong et al., 2019), including the areas of the south of Xinjiang province, the east of Gansu, the northwest of Sichuan province, Qinghai province, and Tibet Autonomous Region (Figure 1). Most regions of the plateau are covered by alpine grasslands, which mainly consist of alpine meadow and steppe (He et al., 2008; Wu et al., 2011), and the grassland ecosystem in this area is very sensitive to the changes of the external environment (Jacobsen et al., 2012). In the sites, the annual mean precipitation decreased from 1,000 to 100 mm from southeast to northwest of QTP, and the annual mean temperature ranged from −5°C in north-western QTP to 20°C in southern QTP during the study period (Sun et al., 2020). The natural gradients and unique combination of water and heat availability across QTP result in various changeable habitats for soil microbes. Therefore, we aimed to detect the changes of microbial homeostasis along the 5,000 km belt-transect survey on the regional scale of QTP.

### Soil and Microbial Properties

For this research, 324 plots from 108 sites (three plots from each site) were sampled from July to August in 2015 the Qinghai–Tibet Plateau, and the selected sample plots along the transect must be natural alpine grasslands and as far away as possible from the interference of human activities. At each site (size of 10 m × 10 m), three paired plots (size of 50 cm × 50 cm) were sorted to obtain the soil samples after the grass above the ground was harvested. Soil samples were excavated by a standard 5-cm-diameter soil auger (at depths of 0–30 cm) in each quadrat. In the laboratory, soil samples were air-dried and sieved through 2-mm mesh as well as ground into fine powder with a ball mill (NM200, Retsch, Haan, Germany).

We used the drying and potentiometric method to measure soil water content (SWC) and the soil pH value (pH), respectively. One subsample was oven-dried at 105°C to a constant weight for measuring soil bulk density (SBD). A vario MACRO cube elemental analyzer (Elementar Analysensysteme GmbH, Germany) was used to measure soil organic carbon (SOC) and soil total nitrogen (STN) (Lu et al., 2015), and the ratio of SOC to STN was expressed as SOC/STN. We obtained SOM by the potassium dichromate oxidation method determining SOC, with universal conversion factor 1.724 (Chen et al., 2018). Moreover, we used the chloroform fumigation method with a universal conversion factor of 0.45 and 0.54 to obtain (MBC) (Joergensen, 1996) and (MBN) (Joergensen and Mueller, 1996), respectively. MBC/MBN was the ratio of MBC to MBN. Furthermore, microbial entropy carbon (MBC/SOC) and microbial entropy nitrogen (MBN/STN), respectively, represent the biologically active fraction (MBC and MBN) and turnover activity of soil C and N pool, reflecting the activity degree of microbial demand for soil C and N resources to form microbial C and N elements (Srivastava and Singh, 1991; Wu et al., 2019).

### Climate Database

We collected the climatic data (daily precipitation and temperature) of 2015 through the Meteorology Information Centre of the Chinese National Bureau of Meteorology^1^, and the data from January to December was calculated to obtain the mean annual temperature (°C) (MAT) and the mean annual precipitation (mm) (MAP) by the average method. Moreover, climatic raster databases were spatially interpolated by the Anusplin 4.2 (Centre for Resource and Environmental Studies, Australian National University, Canberra) with 1 km resolution. The climatic data (MAP and MAT) of our sample sites was extracted from the raster climatic database by ArcGIS 10.2 (ESRI, Inc., Redlands, CA, United States). Additionally, aridity index (AI), an important aridity measure of meteorological factor was calculated (De Martonne, 1926):


$$AI=\frac{MAP}{MAT+10}$$

### Calculation of the Coefficient of Variation and Trade-Off Model

We used the coefficient of variation (CV) to indicate the stability of MBC, MBN, and MBC/MBN in alpine grasslands. CV is the SD divided by the mean value (MN) for the variable (MBC, MBN, or MBC/MBN):


$$CV=\frac{SD}{MN}\times 100\%$$

**FIGURE 2:**
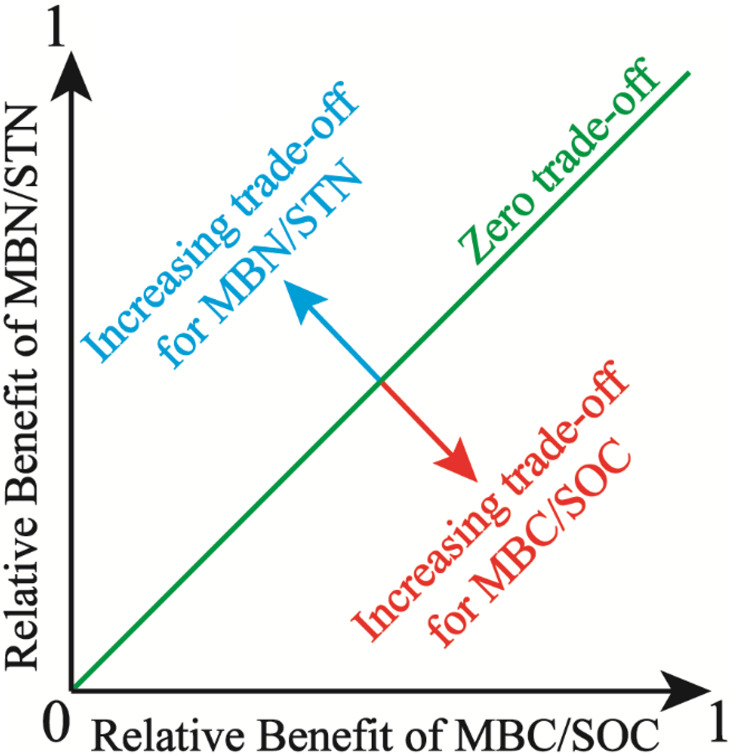
Illustration of the trade-off between microbial entropy carbon (MBC/SOC) and microbial entropy nitrogen (MBN/STN), and the trade-off is calculated as the root mean square error (RMSE) of the individual benefits and increases with distance from the 1:1 line, in which the benefit of MBC/SOC equals the benefit of MBN/STN.

Bradford and D’Amato (2012) established the model for trade-off among individual benefits, which is suitable for living strategies and choices of soil microbes among multiple demand goals (MBC/SOC and MBN/STN). The trade-off model was calculated using the below equations:


$$\left\{ \begin{array}{c} Trade-off=\frac{Benefit_{\left(MBN/STN\right)}-Benefit_{\left(MBC/SOC\right)}}{\sqrt{2}}\\ Benefit_{\left(MBC/SOC\right)}=\frac{{\left(MBC/SOC\right)}_{Obs}-{\left(MBC/SOC\right)}_{Min}}{{\left(MBC/SOC\right)}_{Max}-{\left(MBC/SOC\right)}_{Min}}\\ Benefit_{\left(MBN/STN\right)}=\frac{{\left(MBN/STN\right)}_{Obs}-{\left(MBN/STN\right)}_{Min}}{{\left(MBN/STN\right)}_{Max}-{\left(MBN/STN\right)}_{Min}} \end{array}\right.$$

where (MBC/SOC)_*Obs*_ and (MBN/STN)_*Obs*_ are the observed values of MBC/SOC and MBN/STN, respectively. (MBC/SOC)_*Max*_ (maximum) and (MBC/SOC)_*Min*_ (minimum) are calculated from the entire population of MBC/SOC, the same is also true for MBN/STN. Benefit_(MBC/*SOC)*_ or Benefit_(MBN/*STN)*_ is the magnitude of benefit for individual management objective (MBC/SOC or MBN/STN), being defined as the relative deviation from the mean for a given observation. Moreover, the trade-off between MBC/SOC and MBN/STN reflects soil microbial trade-off strategies about comprehensive demands for soil C and N resources. Specifically, the overall benefit toward MBN/STN is more with higher trade-off value, which implies that soil microbes tend to the turnover of soil N more, which indicates that microbes demand more soil N resource (Chapin et al., 2011). Further, the root mean square error (RMSE) of the Benefit_(MBC/*SOC)*_ or Benefit_(MBN/*STN)*_ was used to quantify the magnitude of the trade-off between MBC/SOC and MBN/STN. The RMSE approximates the average deviation from the mean Benefit_(MBC/*SOC)*_ or the mean Benefit_(MBN/*STN)*_. In two dimensions, the RMSE is the distance from the “1:1 line” of the “zero trade-off” (Bradford and D’Amato, 2012), and the trade-off model is expressed as the conceptual graph (Figure 2).

### Statistic Analysis

The figure of our study area and sample sites was created using ArcGIS 10.2 (ESRI, Inc., Redlands, CA, United States). The one-way ANOVA was used to detect the differences in microbial properties (MBC, MBN, and MBC/MBN), soil properties (SOC, STN, SBD, pH, and SWC), and climatic characteristics (MAP, MAT, and AI) between alpine meadow and steppe. The correlation coefficients were calculated to reveal the relationships between MBC/MBN and environmental factors (MAP, MAT, AI, SOC, STN, SOC/STN, pH, SBD, SWC, and SOM) in alpine meadow and steppe. The above two methods were analyzed by SPSS 19.0 software (SPSS Inc., Chicago, IL, United States). Moreover, SigmaPlot for Windows version 14.0 (Systat Software, Inc., Chicago, IL, United States) were used to generate the relationships of trade-off with environmental factors (MAP, MAT, AI, SOC, STN, SOC/STN, pH, SBD, and SWC) in various grasslands. A linear piecewise quantile regression (LPQR) was used to evaluate the response of trade-off to SOC, STN, and SWC across alpine meadow for the plausible estimation of responses to a given variable (Ruppert et al., 2012). The segment function is calculated as follows:


t1=min⁡(t)

t3=max⁡(t)

$$f1\left(t\right)=\frac{y1*\left(T1-t\right)+y2*\left(t-t1\right)}{T1-t1}$$

$$f2\left(t\right)=\frac{y2*\left(T2-t\right)+y3*\left(t-T1\right)}{T2-T1}$$

$$f3\left(t\right)=\frac{y3*\left(t3-t\right)+y4*\left(t-T2\right)}{t3-T2}$$

f=if(t≤T1,f1(t),if(t≤T2,f2(t),regionf(t)))

## Results

### Variations of Climatic, Soil, and Microbial Variations

**FIGURE 3:**
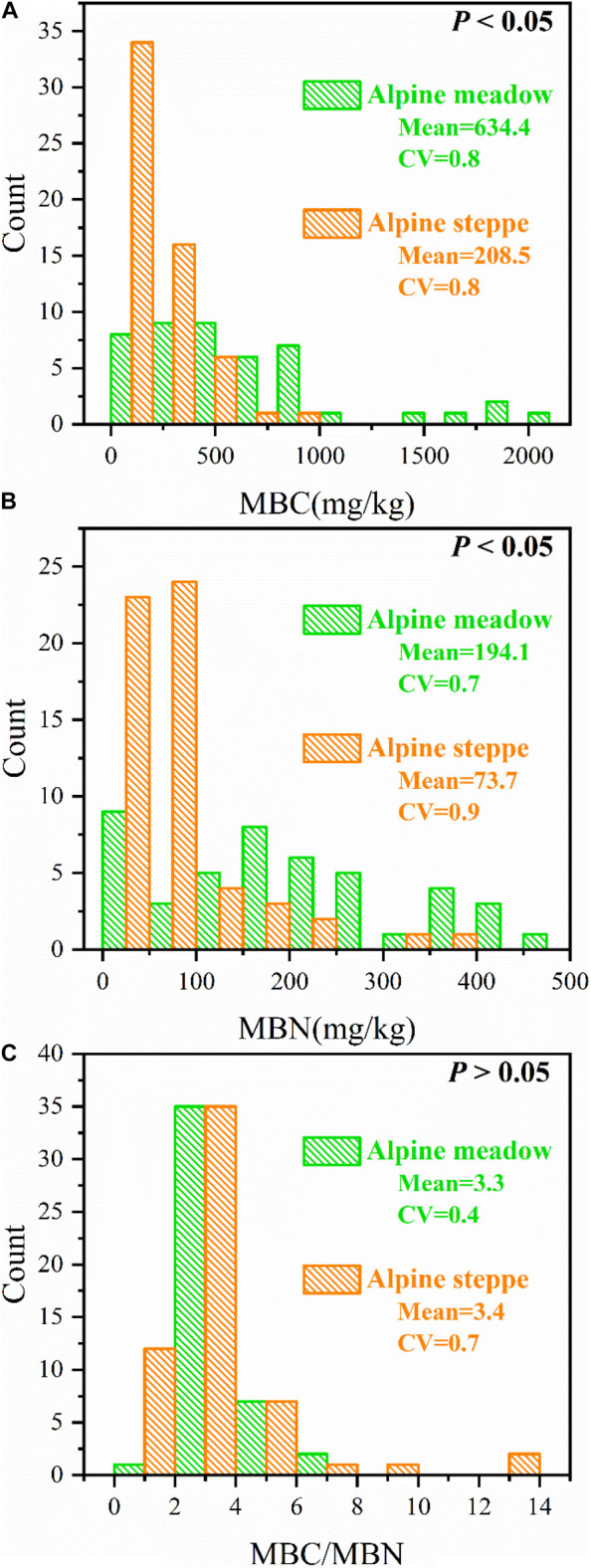
Count distributions of **(A)** soil microbial biomass carbon (MBC), **(B)** soil microbial biomass nitrogen (MBN), and **(C)** the ratio of soil microbial biomass carbon to nitrogen (MBC/MBN) across alpine steppe and meadow. “Mean” and “CV” represent the average values and coefficients of variation of MBC, MBN, and MBC/MBN, respectively. And *P*-values indicate level of significant differences of MBC, MBN, and MBC/MBN between alpine steppe and alpine meadow.

MBC and MBN were entirely different (*P* < 0.05, Figures 3A,B), but MBC/MBN was not significantly different (*P* > 0.05, Figure 3C) between alpine meadow and steppe.

**FIGURE 4:**
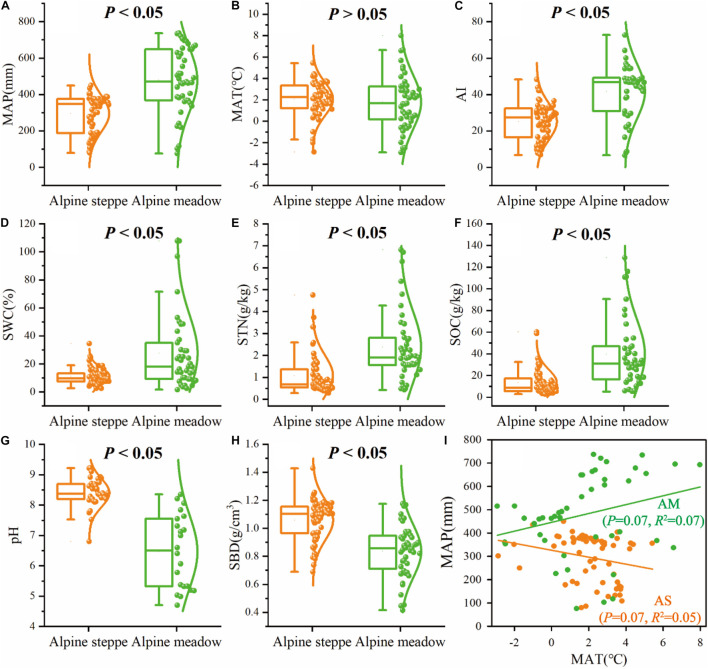
Box-and-whisker plots showing changes in the **(A)** mean annual precipitation (MAP), **(B)** mean annual temperature (MAT), **(C)** aridity index (AI), **(D)** soil water content (SWC), **(E)** soil total nitrogen (STN), **(F)** soil organic carbon (SOC), **(G)** soil pH (pH), and **(H)** soil bulk density (SBD) in alpine steppe (AS) and alpine meadow (AM), *P*-values indicate level of significant differences between alpine steppe and alpine meadow. **(I)** The relationships between MAP and MAT across alpine steppe (AS) and alpine meadow (AM).

The boxplots exhibited significant (*P* < 0.05) differences of MAP, AI, SWC, STN, SOC, pH, and SBD between alpine meadow and steppe (Figure 4). For the mean values of climate (MAP, AI) and soil (SWC, STN, SOC, SBD) variables, MAP ranged from 295.24 to 480.16 mm (Figure 4A), AI ranged from 25.18 to 41.80 (Figure 4C), SWC ranged from 11.21 to 27.67% (Figure 4D), STN ranged from 1.08 to 2.38 g/kg (Figure 4E), SOC ranged from 13.47 to 39.64 g/kg (Figure 4F), and SBD ranged from 1.06 to 0.84 g/cm^3^ (Figure 4H) from alpine steppe to meadow based on regional study of QTP. MAP was negatively correlated with MAT (*R*^2^ = 0.05, *P* = 0.07) in alpine steppe, but was positively correlated with MAT (*R*^2^ = 0.07, *P* = 0.07) across alpine meadow (Figure 4I).

### Homeostatic Analysis of Soil Microbial Biomass Carbon and Nitrogen

The variation coefficient of MBC/MBN in alpine meadow (CV = 0.4) was lower than alpine steppe (CV = 0.7) (Figure 3). Moreover, MBC/MBN was non-significantly (*P* > 0.05) correlated to all environmental factors, and the correlation coefficients ranged between −0.248 and 0.207 in alpine steppe and meadow (Table 1) across regional scale of QTP.

**TABLE 1:** Correlations between the ratio of soil microbial biomass carbon to nitrogen (MBC/MBN) and environmental factors in alpine steppe and meadow; environmental factors include mean annual precipitation (MAP), mean annual temperature (MAT), aridity index (AI), soil organic carbon (SOC), soil total nitrogen (STN), the ratio of SOC to STN (SOC/STN), soil pH (pH), soil bulk density (SBD), soil water content (SWC), and soil organic matter (SOM).

**MBC/MBN**	**Items**	**MAP**	**MAT**	**AI**	**SOC**	**STN**	**SOC/STN**	**pH**	**SBD**	**SWC**	**SOM**
Alpine steppe	Pearson Correlation	0.128	0.046	0.121	–0.019	–0.024	0.004	0.110	–0.057	–0.248	–0.049
	Sig. (2-tailed)	0.317	0.719	0.345	0.888	0.860	0.978	0.519	0.658	0.062	0.714
Alpine meadow	Pearson Correlation	0.128	–0.072	0.141	0.077	0.107	–0.115	0.009	–0.180	0.207	0.027
	Sig. (2-tailed)	0.402	0.638	0.357	0.623	0.495	0.462	0.970	0.243	0.194	0.864

### Trade-Off Between Microbial Biomass Carbon/Soil Organic Carbon and Microbial Biomass Nitrogen/Soil Total Nitrogen

**FIGURE 5:**
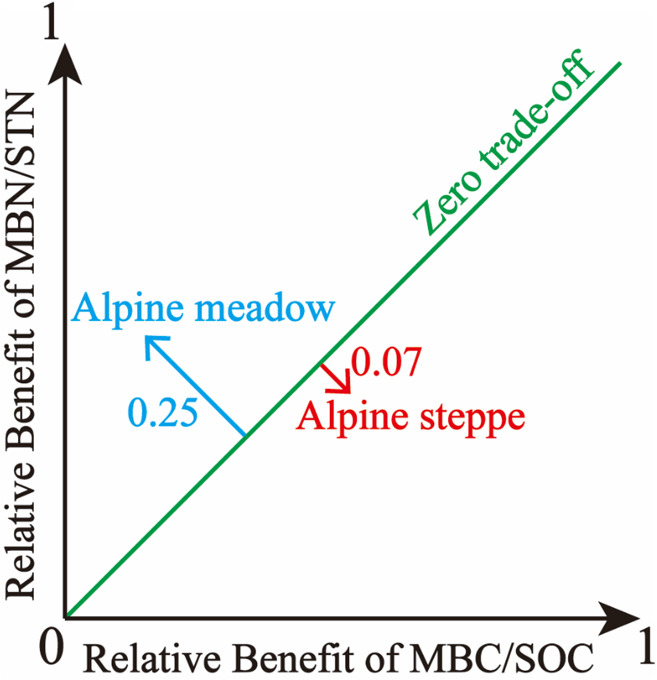
The illustration of overall benefit between microbial entropy carbon (MBC/SOC) and microbial entropy nitrogen (MBN/STN) in alpine steppe and alpine meadow.

**FIGURE 6:**
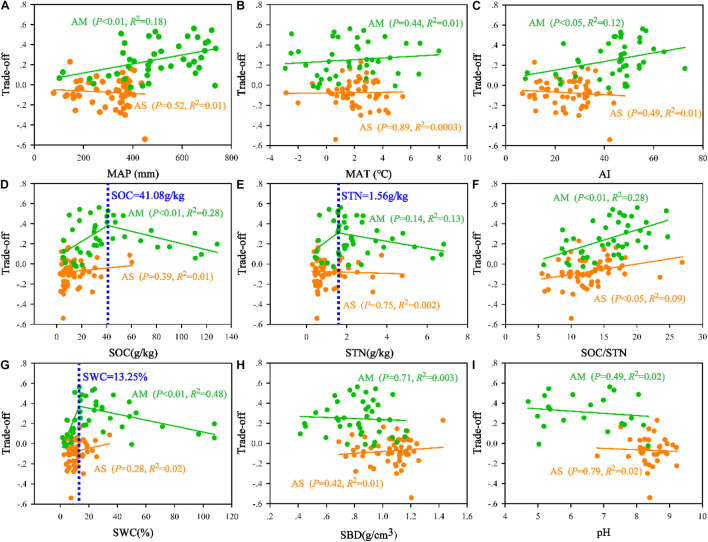
Relationships of the trade-off with **(A)** mean annual precipitation (MAP), **(B)** mean annual temperature (MAT), **(C)** aridity index (AI), **(D)** soil organic carbon (SOC), **(E)** soil total nitrogen (STN), **(F)** the ratio of SOC to STN (SOC/STN), **(G)** soil water content (SWC), **(H)** soil bulk density (SBD), and **(I)** soil pH (pH) in alpine steppe (AS) and alpine meadow (AM).

Based on the trade-off model, the overall benefit was 0.25 toward MBN/STN in alpine meadows and 0.07 toward MBC/SOC in alpine steppes (Figure 5). Moreover, trade-off was positively correlated with MAP (*R*^2^ = 0.18, *P* < 0.01, Figure 6A), AI (*R*^2^ = 0.12, *P* < 0.05, Figure 6C), and SOC/STN (*R*^2^ = 0.28, *P* < 0.01, Figure 6F) in alpine meadows. Trade-off demonstrated unimodality relationships with SOC (*R*^2^ = 0.28, *P* < 0.01, Figure 6D), STN (*R*^2^ = 0.13, *P* = 0.14, Figure 6E), and SWC (*R*^2^ = 0.48, *P* < 0.01, Figure 6G) across alpine meadows ecosystem scale, and all trends of the trade-off were increased and then decreased with increasing SOC, STN, and SWC with the inflection points of 41.08 g/kg (Figure 6D), 1.56 g/kg (Figure 6E), and 13.25% (Figure 6G), respectively. In terms of alpine steppes, trade-off was positively correlated with SOC/STN (*R*^2^ = 0.09, *P* < 0.05, Figure 6F). Trade-off was not significantly (*P* > 0.05) correlated with MAT, SBD and soil pH across alpine meadows and steppes (Figures 6B,H,I).

## Discussion

### More Stable C:N Homeostasis of Microbial Biomass in Alpine Meadows Than Alpine Steppes on the Ecosystem Scale

MBC/MBN almost had no correlation with climate and soil factors in alpine grassland ecosystems (Table 1), indicating that soil microbial biomass stoichiometry was relatively homeostatic meeting the changing environments (Sterner and Elser, 2002; Persson et al., 2010) across both types of grassland. Furthermore, the variation coefficient of MBC/MBN in alpine meadow was lower than steppe (Figure 3C), indicating that stoichiometric homeostasis of soil microbes in alpine meadow is relatively more stable than steppe based on ecosystem scale. Indicators related to water (e.g., MAP, AI, and SWC) and nutrient (SOC and STN) availability in alpine meadow were significantly higher than those in alpine steppe (Figures 4A,C–F), which were vital factors determining different degree of microbial homeostasis. In arid condition (alpine steppe), it is well documented that soil microorganisms could not support constant compound synthesis and their activities of growth, mineralization, and decomposition are constrained with limited soil nutrient and water availability in drought–cold terrestrial ecosystem (Chapin et al., 2011; Kuzyakov and Xu, 2013). Consequently, soil microbes are flexible in their stoichiometry and it is energetically expensive for microbes to maintain the stoichiometric homeostasis under lower water and nutrient availability across alpine steppe (Sterner and Elser, 2002; Mooshammer et al., 2014b). On the contrary, in humid environments (alpine meadow), microbial decomposition and mineralization would be enhanced, and microbes can promote the turnover of soil nutrient. Then it is easier for microbes to balance the uptake of multiple elements under higher water and nutrient availability across alpine meadow (Vandecar et al., 2009; Delgado-Baquerizo et al., 2013; Jilling et al., 2018).

**FIGURE 7:**
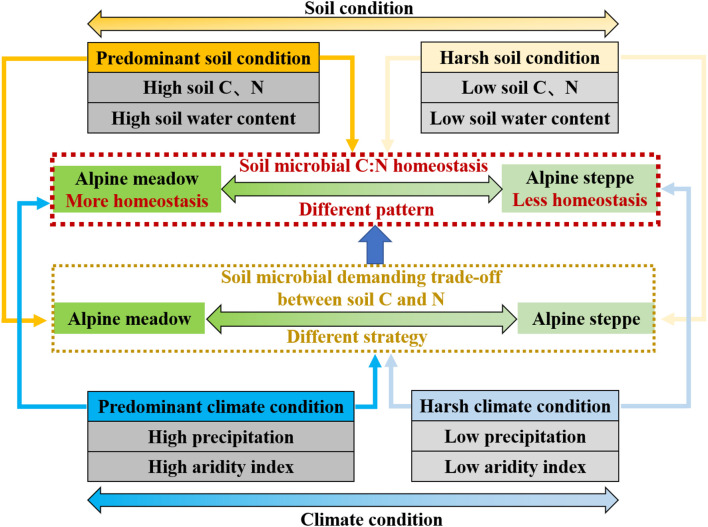
Conceptual model of soil microbial biomass stoichiometry C:N homeostasis in arid (alpine steppe) and humid (alpine meadow) environments. Soil microbes tend to be less homeostatic in their C:N stoichiometry under harsh climate (low precipitation and aridity index) and soil (low soil C and N and low soil water content) condition in alpine steppe. Conversely, soil microbial C:N stoichiometry is more homeostatic under better climate (high precipitation and aridity index) and soil (high soil C and N and high soil water content) condition in alpine meadow. Furthermore, demanding trade-off of soil microbial strategy between soil C and N responding to climate and soil factors is different, leading to different homeostasis pattern of soil microbes between alpine meadow and steppe.

Generally, soil microbes consistently display some changes in body elemental composition in response to variation of soil resources they depend on (Hood and Sterner, 2010; Wang et al., 2018) based on regional scale across QTP in our study. It has been found that the elemental composition between soil resource and soil microbes would be more imbalanced and the growth and utilization efficiency of microorganisms are also lower with worse soil quality. Soil microbes might take non-homeostatic strategy to adjust their element use efficiencies and elemental composition to adapt to harsher environment (Mooshammer et al., 2014b). Given the fact that soil MBC and nitrogen are directly derived from the conversion of soil organic matter and they are very sensitive to variable soil condition (Chapin et al., 2011), soil microbes are likely to be more flexible in their MBC/MBN due to lower soil nutrient availability and harsher climate condition in alpine steppe relative to alpine meadow (Figure 7).

### Using Trade-Off Model to Distinguish Microbial C:N Stoichiometry Homeostasis

Different homeostatic patterns of soil microbial biomass stoichiometry between alpine meadow and steppe reflected that soil microbes respond to the resource they require differently in different habitats (Sterner and Elser, 2002). It has been well documented that the strength of stoichiometric homeostasis is highly relevant to the ecological strategy and adaptability of the organism (Frost et al., 2005; Jeyasingh et al., 2009; Yu et al., 2011), and homeostasis has the ability for consumers (soil microbes) to approach nutritional targets (such as intake or growth) that maximize the fitness in a variable environment region (Raubenheimer et al., 2012; Sperfeld et al., 2017). Therefore, we infer that microbial survival and adaptation strategy of demanding soil C and N resources to form their specific elementary composition of C and N is different in arid (alpine steppe) and humid (alpine meadow) habitat.

Based on the trade-off model (Figure 2), the overall benefit was, respectively, toward MBN/STN and MBC/SOC across alpine meadow and steppe, respectively (Figure 5), indicating that soil microbes tended to the turnover of soil C and N on the ecosystem scale of alpine meadow and steppe, respectively. Furthermore, the trade-off increased with increasing MAP and AI across alpine meadow (Figures 6A,C), indicating that microbial activity increases with more water availability (Mooshammer et al., 2014a) because soil C is richer than soil N for soil microorganisms in alpine ecosystem (Chen et al., 2016). Nevertheless, the increasing water availability (MAP and AI) did not enhance microbial activity in alpine steppe (Figures 6A,C), and that was because of the negative interaction between MAT and MAP (Figure 4I). This indicated that water and heat availability were not synchronous, constraining activities of microbial growth (Melillo et al., 2002; Fierer et al., 2007; Niu et al., 2008). The trade-off increased with increasing SOC and SWC across alpine steppe (Figures 6D,G), which revealed that the limited soil resource intensified the microbial fight for obtaining SOC and SWC. Moreover, SOC/STN had a positive effect on the trade-off across alpine steppe and meadow (Figure 6F) because STN would become a more restrictive nutrient for soil microbes with increasing SOC/STN. According to the law of minimum, organisms respond more strongly to limiting factors, thus soil microbes tended to require more soil N resources to form their own N element with relatively less STN (Chapin et al., 2011).

Notably, as for soil microbes in alpine meadow, the trade-off increased with increasing water and soil properties when SOC, STN, and SWC were lower than the threshold levels of 41.08 g/kg, 1.56 g/kg, and 13.25%, respectively, but decreased when SOC, STN, and SWC were higher than these threshold levels (Figures 6D,E,G), indicating microbial activity for demanding soil nutrient and moisture had weakened with relatively more soil available resources. Moreover, such threshold values for the limitations of water and nutrient availability identified in this study will become a powerful tool for exploring the physiological basis about the trade-off of microbial life history and critical thresholds of alpine ecosystems (Sutherland et al., 2013).

These results suggest that water, heat, and nutrient availability induce changes in microbial trade-off by mediating microbial activity and nutrient turnover (Papanikolaou et al., 2010; Moyano et al., 2013; Serna-Chavez et al., 2013) based on regional study across QTP’s alpine grasslands. Soil microbial strategy of demanding trade-off between soil C and N in response to soil and climate factors was different between alpine steppe and meadow, leading to different variability of soil microbial biomass stoichiometry homeostasis (Figure 7). For soil microbes, stoichiometric homeostatic regulation reflects underlying biochemical allocations and trade-off when they respond to surrounding environments (Hessen et al., 2004) on the QTP’s regional-scale study.

## Conclusion

The soil microbial biomass stoichiometry homeostasis in alpine steppe was relatively less stable than alpine meadow across the regional scale of QTP’s grasslands due to higher water, heat, and nutrient availability in alpine meadow than that in steppe. Meanwhile, the quantified trade-off model is an ideal interpretation to further distinguish stoichiometric homeostasis of soil microbes between alpine steppe and meadow. Based on the trade-off model, soil microbes tended to the turnover of soil C and N across alpine meadow and steppe, respectively. Furthermore, the trade-off significantly increased with increasing SOC/STN, MAP, and AI, and had unimodal relationships with SOC, STN, and SWC across the alpine meadow. In alpine steppe, water (MAP and AI) and nutrient (STN) availability had no remarkable effects on the trade-off. Nevertheless, further studies should be devoted to analyze biogeographic characteristics of homeostasis of the plant on the QTP-regional scale and reveal the effect of biological homeostasis to indicate the structure, function, and stability of alpine ecosystem.

## Data Availability Statement

The original contributions presented in the study are included in the article/supplementary material, further inquiries can be directed to the corresponding author.

## Author Contributions

JF and YY designed the experiment. TL, YL, and YYL sampled and analyzed the data. JF, TL, and YY were involved in drafting the original manuscript. JF and XL were involved in editing and revising the manuscript. All authors read and approved the final manuscript.

## Conflict of Interest

The authors declare that the research was conducted in the absence of any commercial or financial relationships that could be construed as a potential conflict of interest.

## Publisher’s Note

All claims expressed in this article are solely those of the authors and do not necessarily represent those of their affiliated organizations, or those of the publisher, the editors and the reviewers. Any product that may be evaluated in this article, or claim that may be made by its manufacturer, is not guaranteed or endorsed by the publisher.

## Footnotes

1
http://data.cma.cn/
